# Infective endocarditis in Ethiopian children: a hospital based review of cases in Addis Ababa

**DOI:** 10.11604/pamj.2015.20.75.4696

**Published:** 2015-01-28

**Authors:** Tamirat Moges, Etsegenet Gedlu, Petros Isaakidis, Ajay Kumar, Rafael Van Den Berge, Mohammed Khogali, Amha Mekasha, Sven Gudmund Hinderaker

**Affiliations:** 1 Department of Paediatrics and Child Health, Tikur Anbessa University Hospital, Addis Ababa, Ethiopia; 2 Médecins Sans Frontières (MSF), Operetional Research Unit, Luxemburg; 3 International Union Against Tuberculosis and Lung Diseases (The Union), Oslo, Norway

**Keywords:** Ethiopia, children, rheumatic heart disease, systemic embolization, endocarditis

## Abstract

**Introduction:**

Infective endocarditis is an infection of the endocardial lining of the heart mainly associated with congenital and rheumatic heart disease. Although it is a rare disease in children, it is associated with high morbidity and mortality; death due to infective endocarditis has been reported to be as high as 26% in sub-Saharan Africa.

**Methods:**

This was a retrospective review of routinely collected data from patient records.

**Results:**

A total of 40 children (71% female) with 41 episodes of infective endocarditis admitted to a general paediatric ward in Addis Ababa, Ethiopia between 2008 and 2013. Age ranged from 7 months to 14 years, with a median of 9 years (Inter quartile Range: 7-12 years). Rheumatic and congenital heart diseases were underlying risk factors in 49% and 51% of cases respectively. Congestive heart failure, systemic embolization and death occurred in 66%, 12% and 7.3% respectively. Death was associated with the occurrence of systemic embolization (P-value = 0.03).

**Conclusion:**

Rheumatic heart disease was an important predisposing factor for infective endocarditis in Ethiopian children. Late presentations of cases were evidenced by high proportion of complications such as congestive heart failure. A low rate of clinically evident systemic embolization in this study may be a reflection of the diagnostic challenges. High proportion of prior antibiotic intake might explain the cause of significant BCNE. Preventive measures like primary and secondary prophylaxis of rheumatic fever may decrease the associated morbidity and mortality. Early detection and referral of cases, awareness creation about indiscriminate use of antimicrobials, and proper history taking and documentation of information recommended.

## Introduction

Infective endocarditis (IE) is an infection of the internal layer of the heart chambers. It is rare but incidence may be increasing in children [1]. It is associated with high morbidity and mortality. It occurs with underlying rheumatic and congenital heart disease. Africa carries high burden of rheumatic heart disease, which carries a risk of IE and congestive heart failure. Congenital heart disease is more common cause of IE in high income countries [1, 2]. Death rate due to IE, as high as 26% has been reported from sub-Saharan Africa [3, 4]. Early diagnosis, prompt and appropriate treatment of IE is crucial in curing the disease and preventing its serious complications, especially systemic embolization (SE) and death [5, 6]. However, diagnosing IE requires sophisticated procedures [7]. Most important laboratory investigation in the diagnosis of IE is Blood culture. Studies have shown that there is a continuous bacteremia and high rate of positive blood culture in infective endocarditis. In the absence of recent prior antimicrobial therapy up to 95% of cultures positive result were reported for causative microorganism [8, 9]. Studies have shown in a report from low and middle income countries that, blood culture negative endocarditis (BCNE) is in the range of 21-67% [8]. Proper treatment of IE and SE implies administration of expensive antibiotics and cardiac surgery which are rarely available in resource-limited settings [10]. In addition, the costly treatment presents a huge financial burden on both the patients and the health system [11, 12]. In a poor country like Ethiopia, where most health facilities are understaffed, the diagnosis and treatment of IE and SE can be a big challenge and it is likely for cases of IE to be missed [13]. Few published reports are available in children with IE in sub-Saharan Africa. Some of them reported difference in the pattern of IE in children compared with that in the high income countries [14]. Others also reported that IE in children is continuously changing with respect to underlying conditions, predisposing factors, etiologic agents, clinical manifestations, treatment, and outcome [15]. In this paper we describe the clinical profile, treatment outcomes and diagnostic challenges of 41 episodes of IE in 40 paediatric patients admitted and diagnosed by modified Dukes criteria.

## Methods

**Figure 1:**
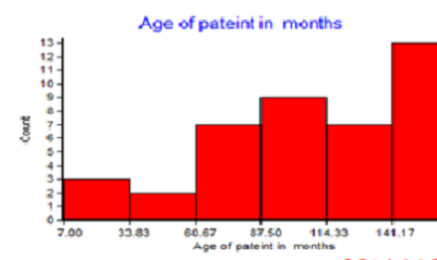
Histogram of age in months of cases of infective endocarditis in children at Tikur Anbessa Hospital in Ethiopia, 2008-2013

**Study design** This was a retrospective study on routinely collected data from patient records during the period of January 2008 to October 2013. **Setting** Ethiopia is located at the horn of Africa and has a population over 90 million. The GNP per capita is only 380 USD. The annual health budget represents 3.6% of the total budget [10]. The health infrastructure consists of various levels from the lowest to the highest: health post, health centre, district hospital, referral hospital and tertiary hospitals. Even though there is an existing referral system between these levels, patient attendance depends on the economic and educational status of the families [11]. This study was conducted at Tikur Anbessa hospital, a tertiary care centre, in Addis Ababa, where IE patients are referred. Therapy continued regardless of negative blood culture result. The cases were referred from all corners of the country. Cases of IE were admitted to the casuality and inpatient ward either from the emergency, regular OPD or paediatric cardiac clinic. The study population were children (0-14 years of age) diagnosed with IE based on modified Duke's criteria (Figure 1). We included confirmed and possible IE cases based on criteria. We excluded cases that did not fulfill the criteria (rejected IE cases). Blood specimen for culture and sensitivity were routinely collected prior to the initiation of anti infective treatment. Sterile procedure used to collect blood sample. 5-10 ml of venous blood is collected and mixed with equal amount of broth. Ideally 3 bottles of specimen are collected. In the lab the specimen are incubated at 37oc. The specimen is checked for turbidity every time to see for growth. The specimens are kept incubated for 14 days in case of infective endocarditis. No special resine is added to remove prior antibiotics from the blood specimen.

Independent variables inquired included: age, sex, date of admission, address, hospital stay, bacteriology, prior antibiotic therapy, type of antibiotic taken priorly and its duration, vegetation on echocardiography, date of diagnosis, date of treatment initiation, type of antibiotic regimen, date of discharge, type of heart lesion, presence of risk factors, cardiac surgery. Outcome variables were IE, systemic embolization, and discharge outcomes. Systemic embolization was referred only to those clinically evident cases. Clinically evident SE to the pulmonaries is considered if the patient develops chest pain, respiratory distress or haemoptysis. Septic SE is considered in a patient, who develops septic shock while he is on treatment for IE. Clinically evident systemic embolization to the central nervous system was defined as, unexplained fever accompanying a stroke in a patient with valvular abnormalities and valvular incompetence [16]. Cure was defined as absence of clinical and laboratory evidence of infection or causative pathogen on blood culture after completion of full course of antimicrobial treatment [17–19]. Vegetations were defined as mobile dense masses attached to the valve or their supporting structure. Data was collected from admission registry book, computer file and patient charts.

### Data analysis

Simple summary statistics was used to describe the cases of IE. Cross tabulation of SE by clinical characteristics was done. Chi-square test was used to test statistical significance of association between SE and clinical characteristics. A p-value < 0.05 was considered as significant. Whenever value below 5 is obtained mid p exact (equivalent of fisher exact value) is used.

### Ethics considerations

This study has been approved by the department research proposal committee of Addis Ababa University, school of health science. This study has met the MSF ethics review board-approved criteria for analysis of routinely collected data. It also satisfies the requirements of the ethics advisory group of the union and meets their approval.

## Results

**Figure 2:**
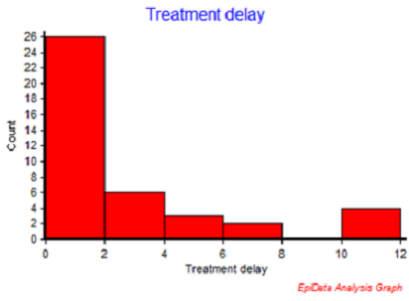
Initiation of treatment in days after admission in cases of infective endocarditis in children at Tikur Anbessa Hospital in Ethiopia, 2008-2013

In this study we reported on a cohort of 41 episodes of IE in 40 children with infective endocarditis (IE) admitted to a general paediatric ward in Addis Ababa between 2008 and 2013. The characteristics of the participants by their clinical profile are shown in Table 1. The age of the participants is displayed in the Figure 1 Systemic embolization occurred in 5 cases (12%; 95% CI 4.6-25.0%). Out of all the cases of IE three cases died, resulting in a case fatality rate for IE of 7.3% (95% CI 1.9% - 18.6%), two of them had a systemic embolization. Note that all the patients had an underlying heart condition, 49% had RHD and 51% had CHD. Figure 2 shows the time from admission to treatment initiation for the patients. A serious condition with congestive heart failure (CHF) was observed in 27 cases (66%; 95% CI 51-79%). Very few had a positive blood culture result (13%). Three-fourth of the cases had taken one or more antibiotics prior to blood culturing. The determinant factors of outcome of cases are shown in Table 2. There were 6 (15%; 95% CI 6.2-28.0%) cases with adverse outcomes, death or systemic embolization, and their clinical profiles are shown in Table 3.

**Table 1:** Clinical characteristics of cases with infective endocarditis in Tikur Anbessa Hospital, Ethiopia, 2008-2013

	Variables	Male	Female	Total No (%)
Age(Yr)	0-7 years	1{2.4%}	7{17.0%}	8{19.5%}
	7 years or more	11{26.8%}	22{53.6%}	33{80.0%}
Underlying disease	CHD	6{14.6%}	15{36.6%}	21{51.2%}
	RHD	6{14.6%}	14{34.1%}	20{48.8%}
Duke's criteria	Confirmed IE	6{14.6%}	12{29.3%}	18{43.9%}
	Possible IE	6{14.6%}	17{41.5%}	23{56.1%}
Risk factors for IE	Yes	1{2.4%}	6{14.6%}	7{16.8%}
	No	4{9.8%}	6{14.6%}	10{24.4%}
	†-Missing information	7{17.1%}	17{41.5%}	24{58.6%}
Prior antimicrobial therapy	Yes	10{24.0%}	22{53.6%}	31{75.6%}
	No	0{0.0%}	1{3%}	1{3%}
	†-missing information			9{21.9%}
Types of antibiotics taken prior to blood culture	Penicillin group	2{6.0%}	10{32.0%}	12{39.0%}
	@-Two or more antibiotics	5{16.0%}	11{35.0%}	17{55.0%}
	Antibiotics not specified		2{6.0%}	2{6.0%}
	Cephalosporin alone			0{0.0%}
Duration of prior antibiotic therapy	1 up to 3 days	1{3.0%}	3{9.0%}	4{13.0}
	4 up to 10 days	4{13.0%}	3{9.0%}	7{23.0}
	More than 10 days	4{13.0%}	14{45.0%}	18{58.0}
	Unknown duration	1{3.0%}	1{3.0%}	2{6.0%}
Blood culture result	&- Positive	2{5.2%}	2{5.2%}	4{13.0%}
	negative	7{22.5%}	20 {64.5%}	27{87.0%}
	†-Missing information			10
Echocardiography	Veg.> = 10mm	5{16.0%}	3{9.6% }	8{25.8%}
	Veg.<10mm	2{6.4%}	8{25.8%}	10{32%}
	No veg.seen	5{16.0%}	8{25.8%}	13{42.0%}
	Missing information			10{24.0%}
Clinically evident SE	yes	1{2.8%}	4{11.0%}	5{14.3%}
	no	9{25.7%}	21{60%}	30{85.7%}
	†-Missing information			6
Congestive heart failure	yes	7{17.0%}	20{49.0%}	27{66.0%}
	No	5{12.2%}	9{21.0% }	14{34.0%}

@ includes-penicillins, cephalosporins, aminoglycosides and antituberculous drugs

& 2 staphylococcus aureus, 2-none typical pathogens for IE

€-blood culture was not done or result missed

† no.of cases with no information out of the total 41 IE episodes

**Table 2:** Potential determinants of outcome among children with infective endocarditis at Tikur Anbessa Hospital in Ethiopia, 2008-2013

		Outcome			
	Variables	Successful outcome n (%)	Adverse outcome no (%)	Total (%)	p-value
Age (years)	0-7 years	7 (88)	1 (13)	8 (100)	P = 1.00
	7 years or more	26 (79)	7 (21)	33 (100)	
Sex	Male	8 (67)	4 (33)	12 (100)	P = 0.20
	Female	25 (86)	4 (14)	29 (100)	
Echocardiography	Veg.> = 10mm1	4 (50)	4 (50)	8 (100)	-
	Veg.<10mm1	8 (80)	2 (20)	10 (100)	
	No veg.seen	11 (85)	2 (15)	13 (100)	
	Missing info	10 (199)	0	10 (100)	
Systemic embolization (SE)	SE present	2 (40)	3 (60)	5 (100)	p = 0.03
	SE not reported	27 (90)	3 (10)	30 (100)	
	Missing info	4	2	6	
Congestive heart failure (CHF)	CHF	22 (81)	5 (19)	27 (100)	p = 1.00
	No CHF	11 (79)	3 (21)	14 (100)	
Treatment delay	Delay < 7 d	30 (83)	6 (17)	36 (100)	p = 0.25
	Delay 7 d or more	3 (60)	2 (40)	5 (100)	
Duration of stay	Less than 28 d	7 (64)	4 (36)	11 (100)	p = 0.18
	28 d or more	26 (87)	4 (13)	30 (100)	

$-Successful outcome-if the patient is discharged improved. Adverse out come,if the patients dies or discharged with major complications

**Table 3:** Demographic and clinical characteristics of cases of infective endocarditis in children with adverse discharge outcomes at Tikur Anbessa Hospital in Ethiopia, 2008-2013

Case	Age (m)	Sex	Underlying heart condition	Risk	Culture	Veg	SE clinical	Duke's	Discharge outcome
1	11	F	CHD + ASD + PHTn	No	S.Aur	No	No	Confirmed	Died
2	120	M	CRVH + SMR + MAR + TR	No	Missing	≥10mm	SE Septic shock	Confirmed	Died
3	144	F	CRHD + MR + TR + PR	Former IE	Neg	<10mm	SE-CNS	Confirmed	Died
4	144	F	CHD + PDA	No	Neg	Multiple	SE-PE	Confirmed	Alive
5	72	F	CHD + PDA	No	Neg	No	SE-CNS	Possible	Alive with hemiplegia
6	84	F	CRVHD + MR + TR	No	Neg	≥10mm	SE-CNS	Confirmed	Alive with hemiplegia

CHD = Congenital Heart Disease; ASD = Atrial Septal Defect; PHT = Pulmonary hypertension; CRHD = Chronic Rehumatic Heart Disease; SMR = Severe Mitral Regurgitation; MI= Mitral Regurgitation; MOA = Moderate Aortic Regurgitation; PDA= Patent Ductus Arteriosus; PR= Pulmonary Regurgitation; SS = Septic Shock; MA = mesenteric arteries; CNS-central nervous sytem,PE-pulmonary embolism

## Discussion

Infective endocarditis in children from all regions of Ethiopia admitted to the tertiary care center in Addis Ababa. There was no published data on pub Med or Google scholar on the topic so far in Ethiopia. Low case fatality rate was noted compared to many reports from elsewhere [20]. Many cases in our study had CHF. Strength of this study is that it represents national data with patients from all over the country. The study also demonstrated the usefulness of strictly standardised Duke's criteria for diagnosis, facilitating comparison of cases. Limitations include lack of positive culture results in most cases, preventing comparison of IE cases by pathogen. Furthermore, as this is a central hospital case series we have little information on the size of the condition at the population level. The pattern of valve involvement and congenital heart disease was not studied in our review. It is undeniable fact that the current data has suffered from missing information, a problem which arise from inadequate history taking and documentation. One might think that there was a selection of admission of less severe cases with better prognoses to the hospital that could be one of the reasons for the fairly good results. It seems likely that the most severe cases had not reached the hospital, but the high proportion of cases with CHF does not seem to support that idea. A relatively low case fatality rate presumably reflects the quality care given once the patient is admitted. There were more girls than boys in our study, and this is also observed in other studies, e.g in Lebanon [21]. The finding may be directly related to female preponderance that is observed in rheumatic heart disease [22].

All the cases of IE had underlying heart disease, and we found that RHD and CHD contributed in equal proportion. This is somehow contrary to the cases in high income countries, where endocarditis in paediatric age group occurs on underlying congenital heart disease and normal heart valves [23]. Our study shows a fairly high proportion of cases with CHF, indicating late admission. Other similar studies in Sub-Saharan Africa show a lower proportion of cases with CHF [21, 24]. Probably an early diagnosis could improve the prognoses, and here alertness among the primary care physician to refer children with heart murmurs for further examination may play an important role. The majority of patients had initiated antibiotics for their IE within 48 hours of admission, which is the aimed limit of guidelines of the ward. This reflects a fairly effective implementation of the diagnostic criteria. A significant proportion initiates treatment at a later time, proving just how challenging the diagnostic process can be [5]. Our study reports a low rate of clinically diagnosed systemic embolization to the central nervous system. A much higher rate was observed in a study in India where 46% of IE cases developed septic embolization to different organs. We think that some seriously sick patients might have died before ever reaching a hospital, and particularly to the child cardiology sections of a tertiary hospital, as arranging transport is a big undertaking from a poor and distant rural settings [25]. As shown in Table 1 Majority of blood cultures did not grow any organism. A similar observation was made by others also in Ethiopia [26].

This is in contrast to most reports even from similar settings from other low or middle income countries [19]. The reason for this observation have to do with the majority of our patient taking antibiotics prior to blood culturing as it is displayed in Table 1. Our finding has been compared to other studies and was found to be much higher [27, 28]. The high proportion of prior antibiotic use in IE cases in the current study may be a reflection of the prevailing indiscriminate use of antimicrobials in our community. Almost all of our patients except few had no surgery. Early surgery in cases of IE prevents the occurrence and recurrence of systemic embolization. Some reports have shown less than 2% recurrence of SE after surgery [29]. Systemic embolization is one of the indications for surgery as part of the management of IE [30]. We already observed significantly higher case fatality among cases with embolization, and this may suggest a tertiary centre like ours should provide these services to improve outcomes.

## Conclusion

Our study showed that rheumatic heart disease is still prevalent in our setting, indicating the necessary preventive measures to decrease the incidence of IE. Systemic embolization is shown to be predictor of death in this study, showing a similar pattern to other reports. Congestive heart failure is the main clinical presentation of IE cases in this study, which may be due to delayed diagnosis. Poor documentation of information, prevalent culture negative endocarditis and lack of surgical intervention for cardiac patients is all observations made in this study. Our study also shows that cases of IE have a fairly good prognosis if hospitalised. This suggests that children with this condition can be successfully saved if detected at an early stage. **Recommendation:** heart murmurs should always alert a primary care physician to refer the patient. IE is often associated in our country with RHD, which is a preventable disease. Doctors should be aware of this risk of rheumatic fever/RHD in children with ordinary streptococcal through infection and treat accordingly, but this is often a diagnostic challenge as viruses often have similar initial symptoms. Future research on IE in low income countries may include multicentre prospective studies. The limitations discussed in this paper should also be addressed. Improving the blood culture isolation technique in the bacteriology laboratory needs special attention and community awareness on indiscriminate use of antimicrobials should be emphasized. Poor history taking and inadequate documentation of information is an important hindrance for conducting meaningful research. Poor dental health is known risk of IE in RHD and CHD and this need to be inquired and documented in the history sheets. Information on the importance of dental health for IE should be communicated to dentists also.
